# Long non-coding RNA H19 promotes corneal neovascularization by targeting microRNA-29c

**DOI:** 10.1042/BSR20182394

**Published:** 2019-05-02

**Authors:** Baoqi Sun, Yiheng Ding, Xin Jin, Shuo Xu, Hong Zhang

**Affiliations:** Department of Ophthalmology, The First Affiliated Hospital of Harbin Medical University, Harbin, Heilongjiang Province, People’s Republic of China

**Keywords:** cornea neovascularization, lncRNA H19, miR-29c, VEGFA

## Abstract

Long non-coding RNA (lncRNA) H19 has been implicated in tumor angiogenesis. However, whether H19 regulates the progression of corneal neovascularization (CNV) is unclear. The present study aimed to determine the function of H19 in CNV and its possible molecular mechanism. Here, we found that the H19 levels were remarkably increased in vascularized corneas and basic fibroblast growth factor (bFGF)-treated human umbilical vein endothelial cells (HUVECs). *In vitro*, H19 up-regulation promoted proliferation, migration, tube formation and vascular endothelial growth factor A (VEGFA) expression in HUVECs, and it was found to down-regulate microRNA-29c (miR-29c) expression. Bioinformatics analysis revealed that H19 mediated the above effects by binding directly to miR-29c. In addition, miR-29c expression was markedly reduced in vascularized corneas and its expression also decreased in bFGF-treated HUVECs *in vitro*. MiR-29c targeted the 3′ untranslated region (3′-UTR) of VEGFA and decreased its expression. These data suggest that H19 can enhance CNV progression by inhibiting miR-29c, which negatively regulates VEGFA. This novel regulatory axis may serve as a potential therapeutic target for CNV.

## Introduction

Corneal avascularity is an indispensable requirement for the cornea to remain transparent and for immune privilege. However, corneal neovascularization (CNV) can occur under conditions of infection, inflammation, immune response, chemical injury, trauma and impaired corneal innervation [1–3], leading to corneal edema, lipid deposition, scar formation, persistent inflammation and a loss of visual acuity [4]. The current therapeutic options for CNV include corticosteroid and non-steroidal anti-inflammatory drugs, anti-VEGF drugs, cryotherapy, photodynamic therapy, laser photocoagulation, fine needle diathermy, and ocular surface reconstruction surgery [3,5]. Unfortunately, the current therapies are unsatisfactory due to their limited effectiveness and undesirable side effects. Therefore, the development of alternative strategies is urgently needed. Recently, some studies have indicated that controlling the expression of non-coding RNAs could help to cure CNV [6–9]. Thus, determining the molecular mechanism of non-coding RNAs in CNV models may be of great significance.

Emerging evidence suggests that non-coding RNAs play a major role in various biological processes, including normal development and disease progression. Non-coding RNAs are a class of RNA molecules without protein-coding potential that includes mainly short non-coding RNAs (snoRNAs, microRNAs (miRNAs), piRNAs etc.) and long non-coding RNAs (lncRNAs). LncRNAs, which are over 200 nucleotides in length, can regulate gene expression in three main ways, including epigenetically, transcriptionally and post-transcriptionally. Notably, lncRNAs can function as endogenous ‘sponges’ that bind miRNAs to regulate miRNA expression and function [10]. Among the many lncRNAs, H19 has been identified to be responsible for tumor angiogenesis [11–14]. In addition, H19 can enhance the angiogenetic capability of mesenchymal stem cells by binding miR-199a and up-regulating vascular endothelial growth factor A (VEGFA) [15], which is a known contributing factor in CNV. However, whether H19 is a potential regulator of CNV progression remains unclear.

MiRNAs, which are a group of short non-coding RNA molecules, can usually bind the 3′ untranslated region (3′-UTR) of a target gene. This action post-transcriptionally regulates the target gene through translational repression or mRNA degradation in a tissue-specific manner. Among all miRNAs, miR-29c has been found to suppress tumor angiogenesis by targeting VEGFA [16]. In addition, based on our prediction using online software (starBase v2.0; http://starbase.sysu.edu.cn/starbase2/index.php), there are potential binding sites between H19 and miR-29c. Thus, we speculated that H19 might promote CNV progression by suppressing miR-29c, which can target VEGFA. To the best of our knowledge, no published studies have explored the relationship between lncRNAs and miRNAs in CNV. Therefore, we explored the function of H19 and miR-29c in CNV progression by *in vivo* and *in vitro* experiments.

## Materials and methods

### Tissue samples

In total, nine vascularized corneas were collected from patients diagnosed with CNV who underwent corneal transplantation at the Ophthalmology Department of the First Affiliated Hospital of Harbin Medical University between February 2018 and September 2018. Nine healthy corneal rims were obtained from the Eye Bank of Heilongjiang Province. The samples were immediately snap-frozen and stored at −80°C until RNA extraction. All patients and donors gave written informed consent prior to participating in the research. This research was carried out in accordance with the World Medical Association Declaration of Helsinki and was approved by the Harbin Medical University Research Ethics Committee (Approval No. 2018107).

### Animal models

All animal experiments were conducted at the Animal Experimental Center of the First Affiliated Hospital of Harbin Medical University and were approved by the Harbin Medical University Animal Ethics Committee (Approval No. 2018003) in accordance with the guidelines of the Association for Research and the Vision and Ophthalmology statement for the Use of Animals in Ophthalmic and Vision Research and the principles of the National Institutes of Health Guide for the Care and Use of Laboratory Animals. A total of 18 female Sprague–Dawley rats weighing 180–200 g were used for the animal experiments. The rats were divided randomly into three groups: a control group, a 7-day group, and a 14-day group. Each group consisted of six rats. A suture-induced rat CNV model was established as previously described [17]. Briefly, under systemic and topical anesthesia, rats received three interrupted sutures in the peripheral corneal stroma with each of the two sutures extending over 120°. The operation was performed only on the right eye of the animals. The corneas were photographed under a slit lamp before the operation and on days 7 and 14 post-operation.

### Cell cultures and transfection

Human umbilical vein endothelial cells (HUVECs) were cultured in Dulbecco’s modified Eagle’s medium (DMEM; HyClone; U.S.A.) supplemented with 10% (v/v) fetal bovine serum (FBS; Biological Industries; Israel), 100 U/ml penicillin, and 100 µg/ml streptomycin (HyClone; U.S.A.) at 37°C in a 5% CO_2_ humidified incubator. The cells were cultivated with different concentrations of basic fibroblast growth factor (bFGF; Peprotech; U.S.A.) for the required time. In addition, pcDNA H19 was purchased from GenePharma (Shanghai, China). Human H19 siRNA (siH19) and the miR-29c mimic/inhibitor were provided by RiboBio (Guangzhou, China). All cell transfections were performed according to the manufacturer’s protocol. Every experiment was repeated for three times independently.

### RNA extraction and real-time PCR

Cells were collected, and total RNA was extracted using TRIzol reagent (Invitrogen; Carlsbad, CA, U.S.A.) according to the manufacturer’s protocol. The RNA concentration was determined by a Nanodrop Spectrophotometer (Nanodrop Technologies; Wilmington, DE). The Bulge-Loop™ miRNA qRT-PCR primer sets were designed and synthesized by RiboBio (Guangzhou, China). Other primers were purchased from Invitrogen (Carlsbad, CA, U.S.A.). The primer sequences are provided in the Supplementary Table S1. Bulge-Loop™ miRNA qRT-PCR Starter Kit (C10210; RiboBio, Guangzhou, China) was used for detection of miRNAs via ABI 7500 Sequence Detection System (Life Technologies; NY, U.S.A.). ReverTra Ace qPCR RT Kit (TOYOBO; Japan) and SYBR-Green PCR Master Mix (TOYOBO; Japan) were used for detection of H19 and VEGFA via ABI 7500 Sequence Detection System (Life Technologies; NY, U.S.A.). The expression of miR-29 was normalized to that of U6 snRNA, while H19 and VEGFA were normalized to GAPDH. The relative expression level was calculated by using the 2^−ΔΔ*C*^_t_ method. Three independently repeated experiments were performed.

### Western blot analysis

Total protein was extracted from cells with RIPA lysis buffer (Thermo Fisher Scientific; U.S.A.) and quantitated with a BCA™ Protein Assay Kit (Beyotime Biotechnology; Shanghai, China). Then, 30 µg of protein was loaded into each well, and the samples were separated on 12% SDS/PAGE gels and transferred on to nitrocellulose membranes (Life Technologies; NY, U.S.A.). Subsequently, the membranes were blocked with 5% non-fat milk (BD Biosciences; U.S.A.) at room temperature for 1 h and incubated with the primary antibody at 4°C overnight. Then, the membranes were washed extensively and incubated with a fluorochrome-labeled secondary antibody (Alexa Fluor 800, Li-COR; U.S.A.) for 1 h at room temperature. Immunoreactivity was detected by an Odyssey fluorescent scanning system (Li-COR, U.S.A.) and analyzed by Image Studio software. GAPDH was used as an internal control. Three independently repeated experiments were performed.

### Cell proliferation assay

HUVECs were seeded in 96-well culture plates at a density of 10000 cells/well after transfection and maintained for 24 h. The cell viability rate was assayed using a Cell Counting Kit-8 (CCK-8, Dojindo; Japan). CCK-8 solution was added to the medium and incubated at 37°C for 2 h. Optical density values were measured at 450 nm. Six replicate wells were established per group, and three independently repeated experiments were performed.

### Wound healing assay

HUVECs were seeded in six-well plates and cultured to 100% confluency after transfection. A sterile pipette tip was used to create a scratch in the monolayer perpendicularly across the center of the well. The floating cells were washed away with warm PBS three times. Images were captured 24 h after wounding under a microscope (Olympus; Japan). Three independently repeated experiments were performed.

### Tube formation assay

The tube formation assay was performed as reported previously [18]. Briefly, Matrigel (50 µl/well; Corning; Bedford, MA, U.S.A.) was applied to a 96-well-plate and placed in an incubator at 37°C for 30 min. Then, HUVECs were seeded on to the gel and maintained for 6 h. Imaging was performed, and the number of branches and the tube length were analyzed by ImageJ software. Three independent repeated experiments were performed.

### Statistical analysis

Significant differences were determined by Student’s *t*test (two-tailed) and one-way ANOVA by using GraphPad Prism software (version 6.0; La Jolla, CA, U.S.A.). The data from each group are presented as the mean ± SEM. *P*-values <0.05 were considered statistically significant.

## Results

### H19 was overexpressed in CNV tissues and bFGF-treated HUVECs

**Figure 1:**
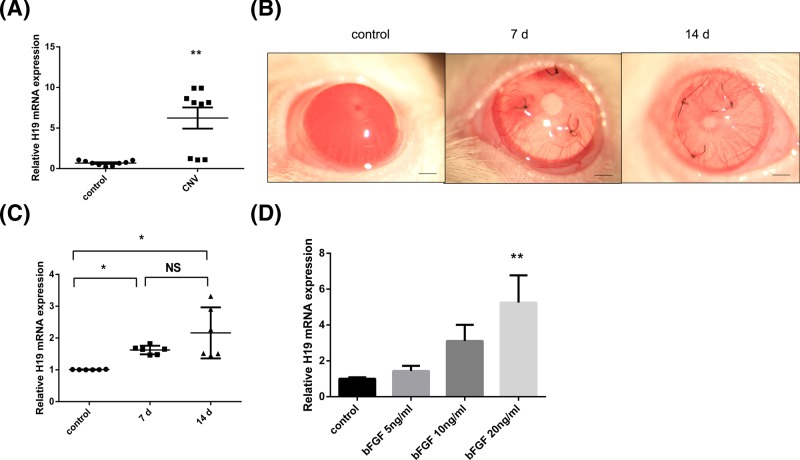
H19 was overexpressed in CNV tissues and bFGF-treated HUVECs (**A**) The relative mRNA level of H19 was significantly higher in human corneal tissues with neovascularization than in matched normal corneas, as determined by real-time PCR (*n*=9). (**B**) The corneas of rats were sutured to induce CNV, and representative images before suturing and 7 and 14 days after suturing are shown. *Scale bar*: 1000 μm (**C**) Relative mRNA level of H19 in rat corneas before suturing and 7 and 14 days after suturing (*n*=6). (**D**) The relative mRNA level of H19 in HUVECs was progressively increased with increasing concentrations of bFGF (0, 5, 10, and 20 ng/ml) (*n*=3). The data are expressed as the mean ± SEM of replicated experiments. *P<0.05, **P<0.01 when compared with the control group. Abbreviation: NS, no significance.

To determine the change in H19 expression during the CNV process, H19 expression in human cornea samples was first measured by real-time PCR. The results showed that the expression of H19 was higher in the CNV group than in the control group (Figure 1A; *P*<0.01). Then, an animal experiment was conducted to further verify this difference in expression. A suture-induced CNV model was chosen, as this is a classic model used to imitate the *in vivo* environment (Figure 1B). The PCR results showed that the level of H19 was higher in the sutured corneas than in the control corneas (Figure 1C; *P*<0.05). However, there was no significant difference in the expression level between the 7-day group and the 14-day group. In addition, a cell experiment was performed to further explore the mechanism of non-coding RNAs in CNV progression. The PCR results showed that the expression level of H19 in the HUVECs progressively increased with increasing concentrations of bFGF; however, the difference was statistically significant at only the 20 ng/ml concentration (Figure 1D; *P*<0.01). Therefore, we used the 20 ng/ml concentration for subsequent experiments. These findings imply that H19 may be strongly correlated with CNV progression.

### H19 promoted proliferation, migration and tube formation in HUVECs

**Figure 2:**
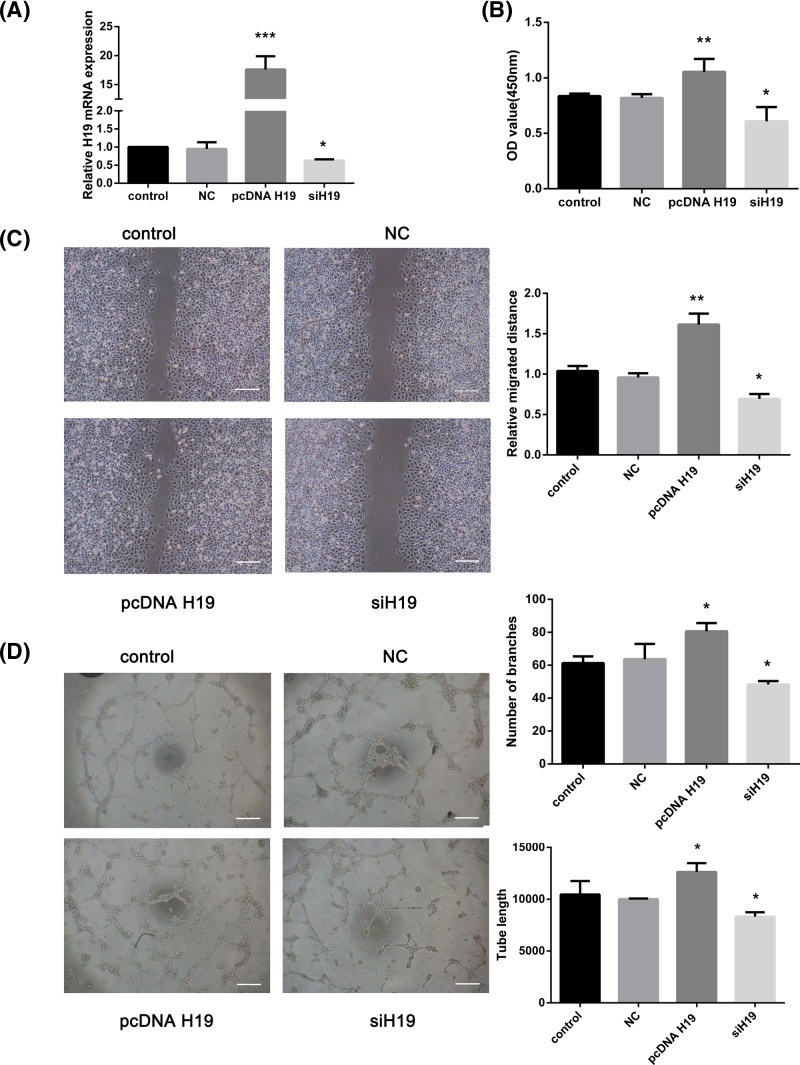
H19 promoted the proliferation, migration and tube formation of HUVECs HUVECs were transfected with pcDNA H19 and siH19. (**A**) Relative mRNA level of H19 as determined by real-time PCR. (**B**) Cell proliferation was determined by CCK-8 assay. Optical density values (450 nm) were quantitated. (**C**) Cell migration was determined by wound healing assay. Representative images are shown, and the relative migrated distance was quantitated. *Scale bar*: 100 μm. (**D**) HUVECs were seeded on Matrigel for the tube formation assay. Representative images are shown, and the number of branches and tube length were quantitated. *Scale bar*: 100 μm. The data are expressed as the mean ± SEM of three independent experiments. *P<0.05, **P<0.01, ***P<0.001 when compared with the control group.

Subsequently, we examined the potential functional role of H19 in HUVECs treated with bFGF. After confirming the transfection efficiency of H19 overexpression and knockdown (Figure 2A), the proliferative capability of HUVECs was measured by CCK-8 assay, the migration capability was assessed by wound healing assay and the angiogenic ability of the HUVECs was analyzed by tube formation assay. As shown in Figure 2B,C, H19 overexpression promoted proliferation and migration in the HUVECs (*P*<0.01), while H19 knockdown decreases these abilities (*P*<0.05). In addition, the results of the tube formation assay showed that the number of branches and the tube length in the pcDNA-H19 group were higher while those in the siH19 group were lower than those in the negative control (NC) group (Figure 2D; *P*<0.05).

### H19 was positively related to the expression of VEGFA in CNV

**Figure 3:**
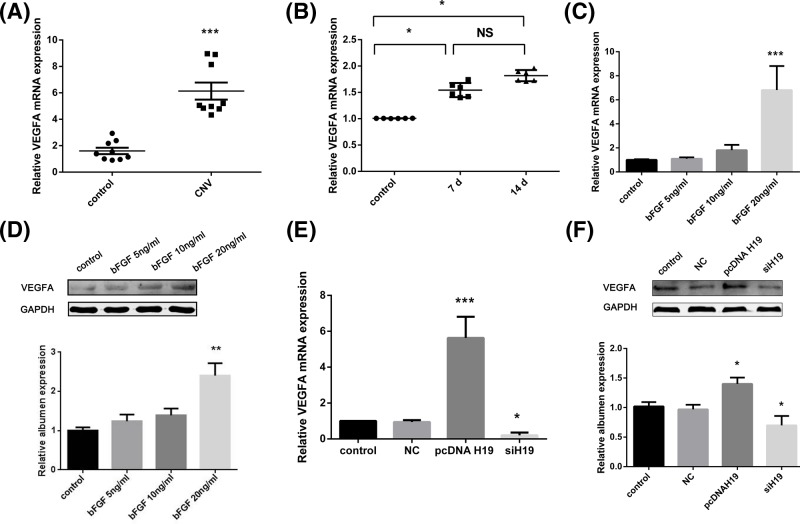
VEGFA expression was regulated by H19 (**A**) The relative mRNA level of VEGFA was significantly higher in human corneal tissues with neovascularization than in matched normal corneas, as determined by real-time PCR (*n*=9). (**B**) Relative mRNA levels of VEGFA in rat corneas before suturing and 7 and 14 days after suturing (*n*=6). (**C**) Relative mRNA levels of VEGFA in HUVECs with increasing concentrations of bFGF (0, 5, 10 and 20 ng/ml). (**D**) Protein levels of VEGFA in HUVECs with increasing concentrations of bFGF (0, 5, 10 and 20 ng/ml). (**E**) Relative mRNA expression level of VEGFA in HUVECs after transfection with pcDNA H19 and siH19 (*n*=3). (**F**) Protein levels of VEGFA in HUVECs after transfection with pcDNA H19 and siH19 (*n*=3). The data are expressed as the mean ± SEM of replicated experiments. *P<0.05, **P<0.01, ***P<0.001 when compared with the control group. Abbreviation: NS, no significance.

A large body of evidence has documented that VEGFA plays an important role in the progression of CNV by contributing to the proliferation, migration and tube formation of HUVECs [19]. Therefore, we attempted to analyze the relationship between H19 and VEGFA in CNV. Consistent with our previous H19 results (Figure 1A,C), VEGFA expression was notably increased in the CNV tissues (Figure 3A; *P*<0.001, B; *P*<0.05); aconcordantly, VEGFA increased with increasing concentrations of bFGF (Figure 3C; *P*<0.001, D; *P*<0.01). Furthermore, we measured the expression of VEGFA after H19 overexpression or knockdown in HUVECs. As shown in Figure 3E,F, VEGFA expression was higher in the pcDNA-H19 group and lower in the siH19 group than in the NC group.

### H19 targeted and negatively regulated miR-29c

**Figure 4:**
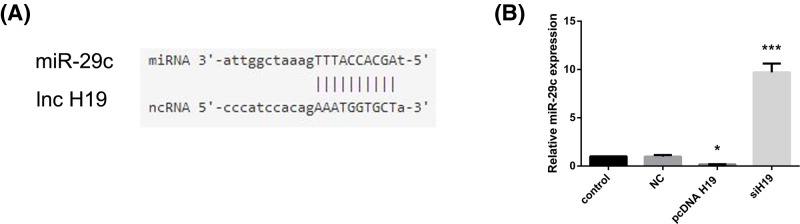
H19 targeted and negatively regulated miR-29c (**A**) Bioinformatics prediction (starBase v2.0) of the putative miR-29c binding sites in the H19 3′-UTR. (**B**) Relative expression of miR-29c after transfection with pcDNA H19 and siH19 determined by real-time PCR. The data are expressed as mean ± SEM of three independent experiments. *P<0.05, ***P<0.001 when compared with the control group.

The above experiments indicated that H19 was positively correlated with VEGFA expression. In addition, lncRNAs are known to function as miRNA sponges to regulate mRNA expression and biological function. To elucidate the mechanism of this regulation, the potential complementary base pairs between lncRNA H19 and miRNAs were examined with bioinformatics software (starBase, v2.0). MiR-29c may be a potential factor in the H19-VEGFA pathway, as it had binding sites for H19 (Figure 4A). Additionally, miR-29c has been previously reported to inhibit the tumor blood supply by targeting VEGFA [16]. Thus, we focused on miR-29c in our research. To elucidate whether miR-29c is regulated by H19, we determined the expression of miR-29c after the overexpression and knockdown of H19 in HUVECs. The real-time PCR results showed that miR-29c expression decreased when H19 was overexpressed (Figure 4B; *P*<0.05), while miR-29c expression increased when H19 was down-regulated (Figure 4B; *P*<0.001).

### MiR-29c expression was decreased in CNV tissues and bFGF-treated HUVECs

**Figure 5:**
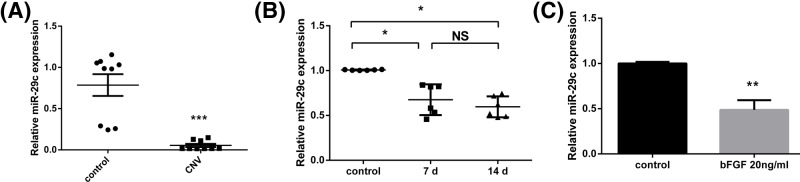
MiR-29c expression was decreased in CNV tissues and bFGF-treated HUVECs (**A**) The relative expression of miR-29c was significantly lower in human corneal tissues with neovascularization than in matched normal corneas, as determined by real-time PCR (*n*=9). (**B**) Relative expression of miR-29c in rat corneas before suturing and 7 and 14 days after suturing as determined by real-time PCR (*n*=6). (**C**) Relative expression of miR-29c in HUVECs before and after treatment with bFGF as determined by real-time PCR (*n*=3). The data are expressed as the mean ± SEM of replicated experiments. *P<0.05, **P<0.01, ***P<0.001 when compared with the control group. Abbreviation: NS, no significance.

Real-time PCR was also used to determine the expression of miR-29c during CNV progression *in vivo* and *in vitro*. The expression of miR-29c was lower in the human CNV group than in the control group (Figure 5A; *P*<0.001). In addition, the expression of miR-29c was markedly lower in the sutured corneas than in the control corneas (Figure 5B; *P*<0.05). However, there was no significant difference in miR-29c expression between the 7-day group and the 14-day group. Furthermore, miR-29c expression in the HUVECs decreased after treatment with bFGF (Figure 5C; *P*<0.01). These results indicate that miR-29c may be involved in CNV progression.

Due to previous report that H19 can bind miR-29a/b in other disease models [11,14,20,21], we also measured the expression of miR-29a/b in rat corneas to validate whether miR-29a/b can mediate H19 function. There was no difference in miR-29a/b expression between the control corneas and sutured corneas (Supplementary Figure S1). This finding suggests that miR-29a/b may be not involved in CNV progression.

### VEGFA is a downstream target of miR-29c in HUVECs

**Figure 6:**
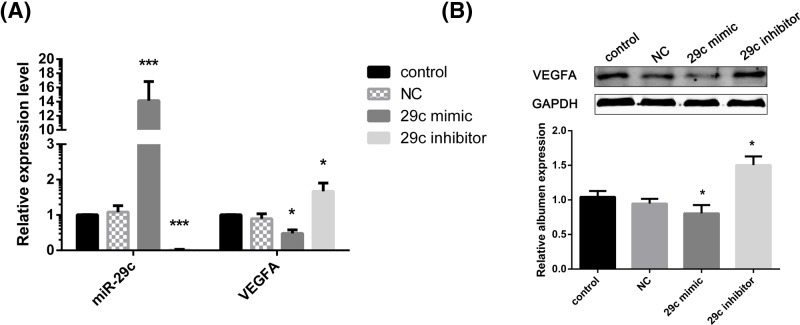
VEGFA was a downstream target of miR-29c (**A**) Relative expression of miR-29c and VEGFA in HUVECs after transfection with miR-29c mimic and inhibitor as determined by real-time PCR. (**B**) Protein levels of VEGFA in HUVECs after transfection with miR-29c mimic and inhibitor as determined by Western blot assay. The data are expressed as the mean ± SEM of three independent experiments. *P<0.05, ***P<0.001 when compared with the control group.

The potential role of miR-29c in CNV was assessed after miR-29c overexpression or knockdown. Real-time PCR was used to validate the efficiency of miR-29c mimic and inhibitor transfection (Figure 6A). The results showed that miR-29c overexpression attenuated the increase in VEGFA caused by bFGF, while the opposite results were observed following miR-29c knockdown (Figure 6A,B; *P*<0.05). Taken together, these results confirm that VEGFA is a downstream target of miR-29c.

### H19 increased the level of VEGFA through miR-29c

**Figure 7:**
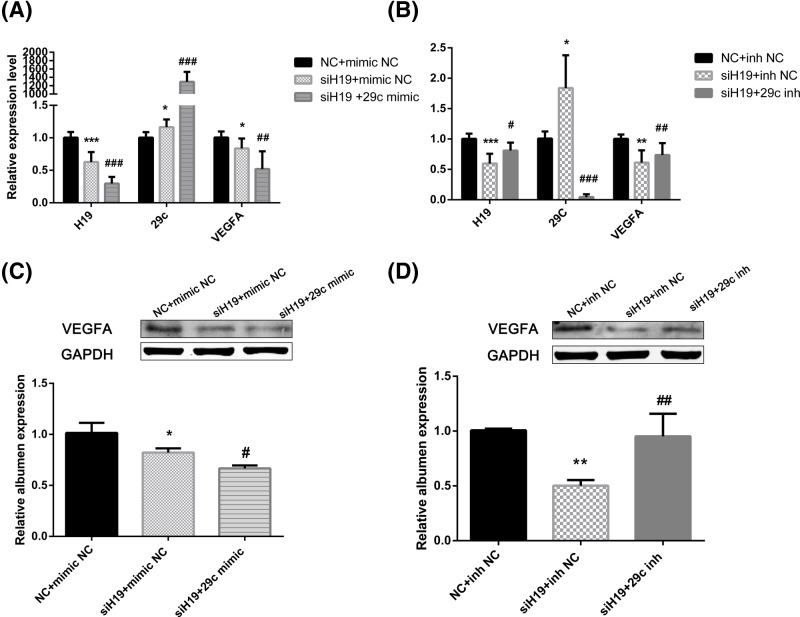
The functional crosstalk of H19, miR-29c, and VEGFA Relative mRNA and protein levels for HUVECs after cotransfection with siH19 and miR-29c mimic/inhibitor. (**A,B**) The relative expression level of miR-29c was visibly increased in HUVECs after transfection with siH19. The relative mRNA level of VEGFA was reduced by the inhibition of H19. This reduction was facilitated by the miR-29c mimic and reversed by the miR-29c inhibitor. (**C,D**) The protein level of VEGFA was in accordance with the mRNA expression results. The data are expressed as the mean ± SEM of three independent experiments. *P<0.05, **P<0.01, ***P<0.001 when compared with the control group. ^#^*P*<0.05, ^##^*P*<0.01, ^###^*P*<0.001 when compared with the siH19+mimic NC/inh NC group.

**Figure 8:**
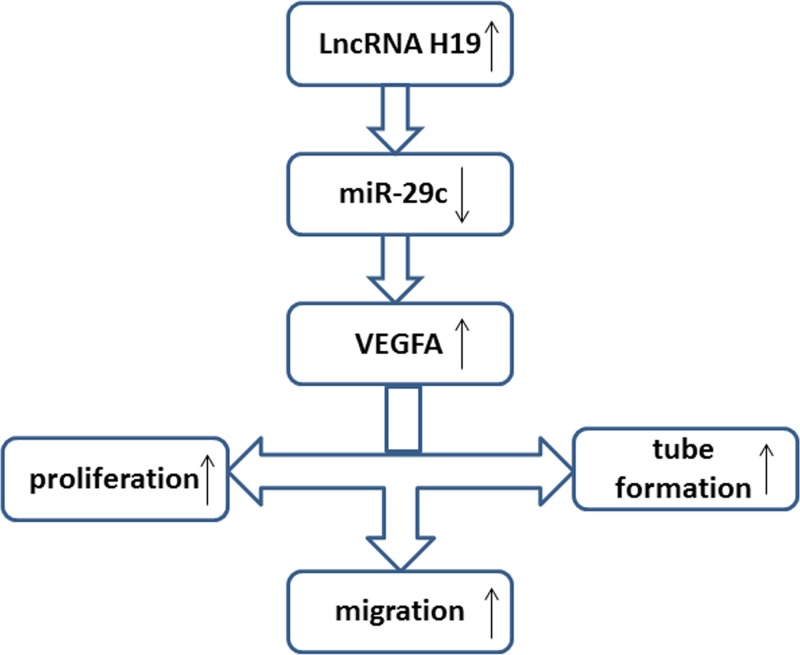
Model diagram of H19/miR-29c/VEGFA regulation in CNV

To further examine the relationship between H19 and miR-29c and between miR-29c and its target VEGFA, HUVECs were cotransfected with siH19 and miR-29c mimics (Figure 7A,C)/miR-29c inhibitor (Figure 7B,D). Then, we assessed H19, miR-29c and VEGFA expression by real-time PCR and Western blot analyses. The real-time PCR results showed that the decrease in H19 expression caused by siH19 could be facilitated by the miR-29c mimic (Figure 7A; *P*<0.001) and reversed by the miR-29c inhibitor (Figure 7B; *P*<0.05). The VEGFA results were similar to the H19 results (Figure 7A–D). Taken together, these findings suggest that miR-29c is an intermediate factor between H19 and VEGFA. The overall conclusion of this study is shown in Figure 8.

## Discussion

In the present study, we found for the first time that the lncRNA H19/miR-29c/VEGFA pathway is involved in corneal angiogenesis. The expression of H19 was increased in vascularized corneas and bFGF-treated HUVECs, and H19 could promote the expression of VEGFA by inhibiting miR-29c in HUVECs. This finding may be one of the underlying mechanisms in CNV progression.

CNV is known to be caused by the decreased expression of anti-angiogenic factors and the increased expression of pro-angiogenic factors, including VEGF, bFGF, transforming growth factor-β, tumor necrosis factor-α, matrix metalloproteinases etc [1]. Of these factors, VEGF, which can exist as VEGFA, VEGFB, VEGFC, VEGFD and placenta growth factor (PlGF), is one of the most important. A very close association between VEGFA and neovascularization has been reported [19]; VEGFA can promote proliferation, migration and tube formation in HUVECs [19]. Hence, we continued to investigate the role of VEGFA in corneal angiogenesis in the present study.

LncRNA H19, which is a maternally expressed gene, is overexpressed in multiple types of cancer, including glioma, bladder cancer, gastric cancer, pancreatic cancer, and cervical cancer, and it plays a role in proliferation, immigration, invasion and angiogenesis [11–14,22–25]. In addition, H19 is closely related to diseases associated with vessels, such as coronary artery disease, acute myocardial infarction and carotid artery injury [26–28]. H19 is also expressed at high levels in embryonic trophoblast tissue and secretory endometrial tissue, which are sufficiently vascularized to support the embryo and repair the uterine vascular bed [29–31]. Taken together, this evidence suggests that H19 is involved in angiogenesis under both physiologic and pathologic conditions. In our study, the H19 levels were significantly higher in vascularized corneas and bFGF-treated HUVECs than in control corneas and cells. In addition, H19 can promote proliferation, migration, tube formation and VEGFA expression in HUVECs. Thus, H19 and CNV progression seem to be closely associated. However, the specific mechanism of lncRNA H19 in the development of CNV remains unclear, and whether miRNAs are involved in this mechanism still needs to be investigated.

A number of studies have implicated miRNAs in the process of angiogenesis; for example miR-210 [32], miR-126 [33] and miR-31 [34], play roles in promoting angiogenesis, and miR-184 [18,35], miR-150 [36], miR-21 [37], miR-29c [16], miR-221/222 and miR-424 [38–40] have a suppressive effect on angiogenesis. MiRNAs, such as miR-93, have also been reported to have varied effects in different tissues [41,42]. Notably, miR-29c has been reported to be closely related to the process of angiogenesis; miR-29c can suppress the proliferation, migration and angiogenic capabilities of HUVECs [43]. In addition, miR-29c can function as a tumor suppressor by targeting VEGFA and inhibiting the tumor blood supply [16]. Our study found that the expression of miR-29c was reduced in patients and animals with CNV, and these results were consistent with the results observed in bFGF-treated HUVECs. Furthermore, miR-29c could negatively regulate VEGFA. This relationship has been further confirmed by other researchers in other disease models [16].

Emerging studies have shown that there is functional cross-talk between lncRNAs and miRNAs, but the role of their interaction in the progression of CNV remains unknown. Previous studies have shown that H19 can bind both miR-29a and miR-29b in other disease models [11,14,20,21]. We also measured miR-29a/b expression in our experiment. The miR-29a/b expression level remained unchanged after suturing. This finding suggests that there may be no association between miR-29a/b and CNV. In addition, through prediction using bioinformatics software, we found that miR-29c can interact with H19. Because there is a high degree of sequence homology between miR-29b and miR-29c and because the binding sites between H19 and miR-29b or miR-29c are the same [44], we conclude that miR-29c also interacts with H19. In this study, the level of miR-29c was significantly increased after H19 inhibition. Thus, H19 can bind miR-29c and inhibit its expression, resulting in increased levels of the miR-29c target gene VEGFA. In addition, we found that a miR-29c inhibitor could reverse the accelerative effects of H19 *in vitro*. These results imply that H19 can promote VEGFA expression and angiogenic capability at least partially through the miR-29c/VEGFA axis.

Although we know the potential role of H19 in the pathogenesis of CNV, we still have no idea what factors triggered the increase in H19 expression in CNV. Klein et al. [45] reported that cofactors of LIM domain proteins (CLIMs) could bind to estrogen receptor α to regulate H19, which can influence corneal epithelial cell proliferation and the expression of some adhesion genes. This raises the possibility that CLIMs dysfunction in the cornea could also impact H19 expression in CNV, including in corneal cells and vessel endothelial cells. This hypothesis still needs further research in the future. As we found in our research, H19 was overexpressed in vascularized corneas. Thus, H19 overexpressed was induced when CNV occurred. To some extent, increased H19 could be considered as an outcome of CNV. In addition, H19 could also promote the expression of VEGFA by targeting miR-29c, which resulted in CNV progression. This finding implies that H19 could facilitate CNV progression. Therefore, it seems that increased H19 was not only the outcome of CNV but also one of the causes of CNV progression. These two activities promote each other.

Several studies have reported that the promotion or inhibition of non-coding RNAs helps to decrease CNV to some extent. Mulik et al. [6] and Bhela et al. [7] reported that CNV could be obviously diminished by the local administration of miR-132 and miR-155 antagomir nanoparticles in herpetic stromal keratitis (HSK) animal models. Zhang et al. [8] also reported that CNV progression could be inhibited and that VEGF expression could be reduced by subconjunctival injections of miR-204 agomir in sutured animal corneas. In addition, an miRNA mimic has been used in human clinical trials to treat liver cancer [46]. These reports indicate that the application of non-coding RNA mimics/inhibitors may be a novel therapeutic strategy for CNV in the future. Further animal transfection experiments still need to be performed to verify our conclusions.

There were also other limitations to this research. Because it is difficult to collect human corneal tissues, it could be beneficial to verify our conclusions in studies involving more samples. In addition, we examined the changes in only sutured animal corneas; it may be necessary to examine the changes in other well-established CNV models, such as those involving cornea alkali burns. In addition, we chose an immortalized HUVEC lines instead of primary human corneal cells, which is another limitation. Thus, additional studies should be performed in the future.

## Conclusion

In summary, the present research revealed for the first time that lncRNA H19 was expressed at high levels and that miR-29c was expressed at low levels in vascularized corneas. We also demonstrated that lncRNA H19 enhances angiogenic capability by acting as a molecular sponge of miR-29c to regulate the expression of VEGFA *in vitro*. The present study may aid in elucidating a potential therapeutic target for corneal angiogenesis in the future.

## Supporting information

**Supplementary Figure S1:** 

**Supplemental Table S1:** The primer sequences are shown below.

## Abbreviations

bFGF: basic fibroblast growth factor
CCK-8: Cell Counting Kit-8
CLIM: cofactor of LIM domain protein
CNV: corneal neovascularization
HUVEC: human umbilical vein endothelial cell
lncRNA: long non-coding RNA
miRNA: microRNA
NC: negative control
VEGFA: vascular endothelial growth factor A
