# Supplementary material for: Long non-coding RNA H19 promotes corneal neovascularization by targeting microRNA-29c
Source: Biosci Rep. 2019 May 2;39(5):BSR20182394. doi: 10.1042/BSR20182394 (PMC6499455; doi:10.1042/BSR20182394)
Supplement: Supplementary file 1 [file bsr20182394_Supp1.pdf]

Supplemental Figure S1

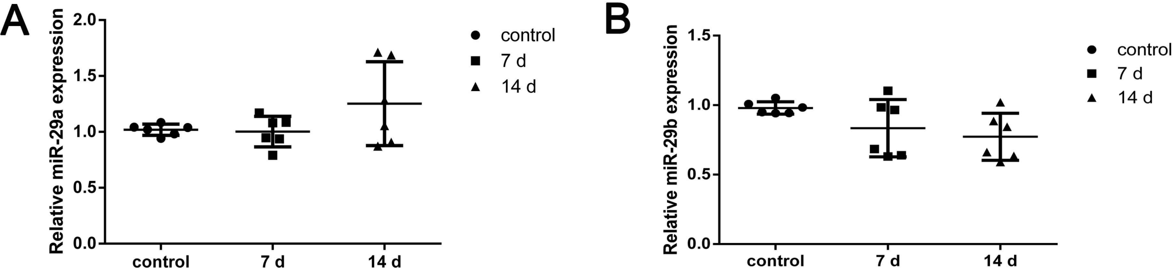

Supplemental Table S1 The primer sequences are shown below.

GAPDH (hsa/rno)	F	5’-AAGAAGGTGGTGAAGCAGGC-3’
	R	5’- TCCACCACCCTGTTGCTGTA-3’
H19 (hsa)	F	5’-ATCGGTGCCTCAGCGTTCGG-3’
	R	5’-CTGTCCTCGCCGTCACACCG-3’
H19 (rno)	F	5’-TTGAAAGAGCAGACCCACACA-3’
	R	5’-CAGGAAAGGAGGAAGAAGAAAA-3’
VEGFA (hsa)	F	5’-GTATTTGACTGCTGTGGACTTGA-3’
	R	5’-GGCTGGGTTTGTCGGTGT-3’
VEGFA (rno)	F	5’- AGAAACCCAATGAAGTGGTG-3’
	R	5’-ACTCCAGGGCTTCATCATTG-3’
U6 (hsa/rno)	F	5’-CTCGCTTCGGCAGCACATATACT-3’
	R	5’- ACGCTTCACGAATTTGCGTGTC-3’
